# 585. Development of a Monitoring System for Quality Assurance of Antibiotic Susceptibility Testing in a Microbiology Laboratory

**DOI:** 10.1093/ofid/ofad500.654

**Published:** 2023-11-27

**Authors:** Gabriel Fang Yih Lim, Wendy Bee Leng Lee, Kar Mun Lee, Yuan Shen Low, Kang Ren Ng, Iris Sing Pei Lim, Huina Yang, Jonathan Chia

**Affiliations:** Tan Tock Seng Hospital, Singapore, Singapore, Singapore; Tan Tock Seng Hospital, Singapore, Singapore, Singapore; Tan Tock Seng Hospital, Singapore, Singapore, Singapore; Tan Tock Seng Hospital, Singapore, Singapore, Singapore; Tan Tock Seng Hospital, Singapore, Not Applicable, Singapore; Tan Tock Seng Hospital, Singapore, Singapore, Singapore; Tan Tock Seng Hospital, Singapore, Singapore, Singapore; Tan Tock Seng Hospital, Singapore, Singapore, Singapore

## Abstract

**Background:**

Antibiotic susceptibility testing (AST) procedures are sensitive to variations in testing conditions. Our diagnostic laboratory is compliant with recommendations set out in Clinical and Laboratory Standards Institute (CLSI) M100 (33^rd^ edition) and M02-A12 (12^th^ edition). A perceived increase in weekly quality control (QC) failures prompted the development of an electronic monitoring system for quality assurance of antibiotic susceptibility testing.

**Methods:**

Zone size data for each antibiotic-organism QC combination were entered on Microsoft® Excel®, and formatted to generate graphs. The data were collected for at least 6 months before and after implementation. This monitoring system allowed the observation of trends which prompted further reviews of laboratory processes.

**Results:**

Persistent recurrent borderline results reflected on the monitoring system sparked the implementation of systematic investigations into QC failures; a replacement program for QC strains; and standardization of incubation timings and desiccant usage. Using new American Type Culture Collection (ATCC) reference strains reduced the percentage of QC antibiotic-organism data which failed or were borderline, and led to greater consistency in testing (see Figure 1). Visual presentation of QC data triggered greater reflection. For instance, recurrent failures of ertapenem disks against *Pseudomonas aeruginosa* ATCC 27853 led to acknowledgement from the manufacturer that the loss of disk potency was due to the shipment of disks nearing the end of shelf life (see Figure 2). The trends in cefepime zone sizes against *P. aeruginosa* ATCC 27853 also prompted an internal review and these improved when antibiotic disk dispensers were consolidated to improve disk utilization (see Figure 3).

**Figure:**
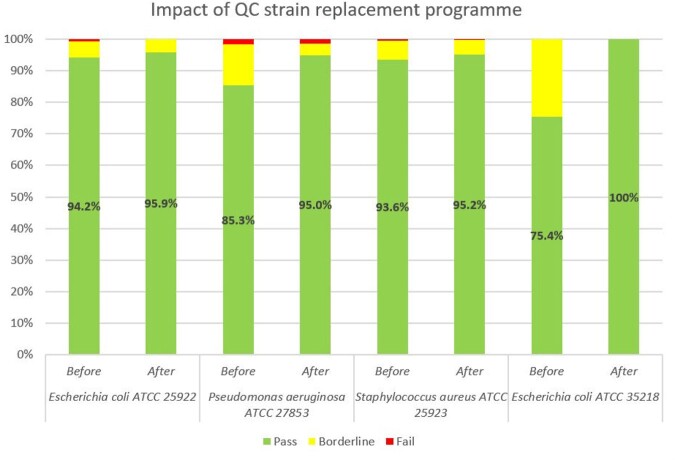

**Figure:**
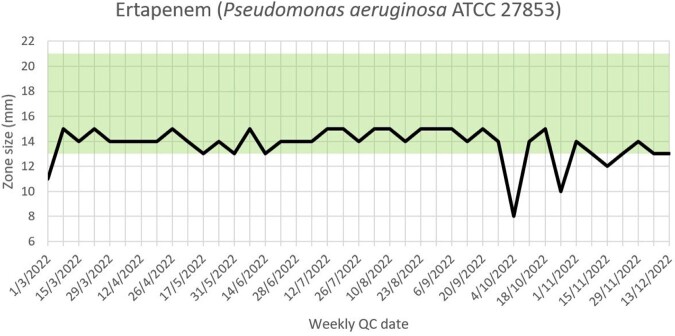

**Figure:**
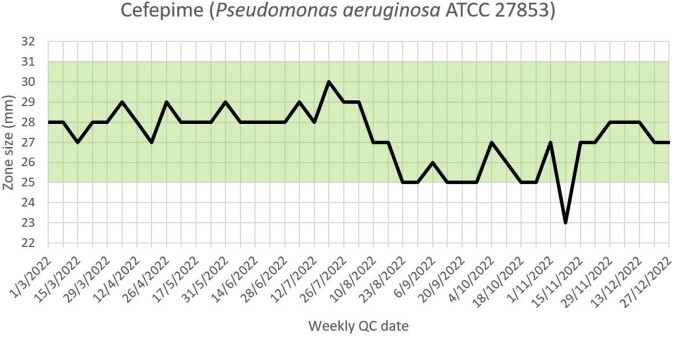

**Conclusion:**

Systemic monitoring of QC trends is low cost and highlights issues which were previously not apparent. Standardization of practices and a systematic troubleshooting process have improved the quality of our disk diffusion susceptibility testing.

**Disclosures:**

**All Authors**: No reported disclosures

